# Origins, Importance and Genetic Stability of the Prototype Strains Gilliam, Karp and Kato of *Orientia tsutsugamushi*

**DOI:** 10.3390/tropicalmed4020075

**Published:** 2019-04-30

**Authors:** Daryl J. Kelly, Paul A. Fuerst, Allen L. Richards

**Affiliations:** 1 Viral and Rickettsial Diseases Department, Naval Medical Research Center, Silver Spring, MD 20910, USA; allen.richards@comcast.net; 2 Department of Evolution, Ecology and Organismal Biology, The Ohio State University, Columbus, OH 43210, USA; fuerst.1@osu.edu; 3 Department of Preventive Medicine and Biostatistics, Uniformed Services University of the Health Sciences, Bethesda, MD 20814, USA

**Keywords:** *Orientia*, scrub typhus, Gilliam, Karp, Kato, *O. tsutsugamushi*, comparative genomics, genetic stability, serogroups

## Abstract

Scrub typhus, a chigger-borne febrile illness, occurs primarily in countries of the Asia-Pacific rim and islands of the Western Pacific. The etiologic agent is the obligate intracellular rickettsial bacterium *Orientia tsutsugamushi*. Research on *O. tsutsugamushi* has relied on the availability of several prototype strains, which were isolated from human cases of scrub typhus in the 1940s and 1950s. We review the history of the three original, and most important, prototype strains, Gilliam, Karp and Kato, including information on their isolation, their culture history, their clinical characteristics, their importance within the research literature on scrub typhus, and recent advances in elucidating their molecular genomics. The importance of these strains to the research and development of clinical tools related to scrub typhus is also considered. Finally, we examine whether the strains have been genetically stable since their isolation, and whether prototype strains maintained in separate laboratories are identical, based on pairwise comparisons of several sequences from four genes. By using genetic information archived in international DNA databases, we show that the prototype strains used by different laboratories are essentially identical, and that the strains have retained their genetic integrity at least since the 1950s. The three original prototype strains should remain a standard by which new diagnostic procedures are measured. Given their fundamental position in any comparative studies, they are likely to endure as a critical part of present and future research on scrub typhus and *Orientia*.

## 1. Introduction

Scrub typhus is a chigger-borne febrile illness of humans, caused by the rickettsia *Orientia tsutsugamushi*. The disease occurs primarily in countries of the Asia-Pacific rim and islands of the Western Pacific, an area often referred to as the “tsutsugamushi triangle,” roughly bordered by northern Japan and far eastern Russia in the north, to northern Australia in the south, and to Pakistan and Afghanistan in the west [1,2]. More recently, cases with scrub typhus or scrub typhus-like disease have been described in a wider geographic area, including areas much further east in south Asia, and into Africa, as well as in South America [2,3,4,5]. Some of these represent newly recognized agents closely related to *O. tsutsugamushi*, such as “*Candidatus* Orientia chuto” [4]. Although from the early 20th-century, scrub typhus was recognized and reported locally, it was not until the 1920s that the agents of various typhus-like rickettsial diseases began to be clearly differentiated [6,7]. For scrub typhus, this was due in part to the introduction of the Weil–Felix test and particularly to the fortuitous discovery in 1926 of the OX-K (Kingsbury) strain of *Proteus mirabilis* as a serological antigen [6]. This antigen differentiates scrub typhus from typhus cases caused by other rickettsiae (i.e., epidemic or louse-borne typhus, caused by *Rickettsia prowazekii,* or endemic, murine or shop typhus associated with *R. typhi*, formerly referred to as *R. mooseri*). The differentiation occurs because antibodies against OX-K specifically result in agglutination in the presence of antigens in the serum from scrub typhus-convalescent patients [6,8,9]. The use of OX-K provided the first clear indication that scrub typhus could be distinguished serologically as a disease separate from other typhus forms. Although now considered less accurate than other techniques, such as the indirect fluorescent antibody test (IFA), the Weil–Felix test has been used to differentiate the rickettsial diseases for over one hundred years and was well respected in its day. For research on any infectious disease to proceed, standardization of research material must be accomplished. Often this occurs when researchers unconsciously utilize the same strain or isolate as the subject of their analyses. This is often driven simply by the availability of biological material for study. Ultimately, this appears to have been the case for scrub typhus. 

The original isolation of the agent of tsutsugamushi disease was accomplished in the late 1920s in Japan, with the most credible evidence suggesting that Mataro Nagayo of Tokyo Imperial University first correctly identified the agent of scrub typhus, and successfully grew it in rabbit cell culture, naming it *Rickettsia orientalis* [10,11,12]. None of the cultured material from pre-World War II (WWII) Japan appears to have survived the war, with one possible exception [13]. Work was also being performed in British Malaya, where William Fletcher and Raymond Lewthwaite and colleagues had used the Weil–Felix test to separate scrub typhus from other rickettsial diseases [6]. Fletcher reported unsuccessful attempts to infect laboratory animals with material from scrub typhus as early as 1925 [14]. Lewthwaite and S.R. Savoor, using material from over 100 patients, tried unsuccessfully during the early 1930s to experimentally infect guinea pigs with the scrub typhus agent, and maintain infections in serial transfers [15]. In 1932, they succeeded with a single isolate, the Seerangayee isolate [16]. A second strain, the Raub strain, was isolated by intra-ocular inoculation in rabbits [16]. By 1936, these two isolates had been maintained through almost 100 transfers [16]. Rapmund [8] indicated that Lewthwaite was ordered out of Malaya prior to the Japanese invasion “so that he could carry strains of Malayan scrub-typhus rickettsiae to Great Britain for vaccine development.” The effort became known as “Operation Tyburn” and was directed by the Wellcome Foundation. This cotton rat lung-derived vaccine was tested immediately after the war but found to be ineffective [9]. 

World War II played a significant role in expanding our knowledge of scrub typhus [17] (as reviewed in the next section). Military medicine in WWII was responsible for the isolation and study of several scrub typhus rickettsial strains of human origin obtained using experimental infection of mice, including the Gilliam strain [12,18], or of guinea pigs, including the Karp strain [19]. Most of the strains of human origin obtained during WWII have not been studied extensively in over 50 years, although a number remain stored in culture collections. In the years following WWII, the Seerangayee and Raub isolates from Malaya, isolated prior to WWII, were used by several investigators in various comparative studies, especially on antigen cross-reactivity [18,20,21,22], susceptibility to antibacterial compounds [23,24,25], or studies of pathogen persistence [26]. However, no references could be found that would indicate that either of these strains was used in any study after 1949, despite being present in culture collections in Australia and the U.S. at least through the early 1950s. Other strains that were isolated during WWII, such as Kostival, Imphal and Volner, also appear in some studies before 1950, but appear only very sporadically in later studies. In contrast, two isolates, Gilliam and Karp, emerged from World War II as important foci of research. Together with a third isolate, the Kato strain, obtained in the early 1950s, they played a central role in subsequent studies of the organism and the disease.

We have previously [1] used the term “prototype strain” to refer to an early isolate that has been used as a reference for the characterization of other subsequent isolates of an organism. Prototype strains are important because they can be used to provide baselines for experimental studies, such as studies of pathogenicity, immunological responses, vaccine production, etc., and can be used to produce reagents for use in diagnosis. As such, the three strains Gilliam, Karp and Kato can be considered the original prototype strains for the study of scrub typhus. Historically, Shishido was the first to refer to the Gilliam, Karp, and Kato strains as prototype strains [27,28]. Since then, many authors have also recognized these three strains as representing prototypes [29,30,31,32,33,34,35,36]. Other authors also referred to the three strains as prototypes, but added some additional, more recently isolated, strains as prototypes of alternative serogroups [37,38,39,40,41]. 

The three original prototype strains were analyzed in ways that contributed substantially to our knowledge of the taxonomic relationship of the agent of scrub typhus to other rickettsiae. They were used in the first molecular comparisons between scrub typhus isolates and rickettsial taxa in the 1990s [42,43,44]. These genetic investigations concluded that the agent of scrub typhus was significantly divergent from members of the genus *Rickettsia*, and a new genus for the scrub typhus agent, *Orientia*, was proposed [45]. The prototype strains have subsequently been important in providing a phylogenetic basis for the comparison of isolates of scrub typhus [1,46].

The continued use of the established prototype strains in research and product development for intracellular bacteria such as *Orientia* is especially important. Prototype strains accrue importance, because their culture has been well documented, and their use standardizes information from different studies, whether that study is directly of the prototype strain, or is a comparison of the prototype with a newly isolated culture. Here, we review the history of the three original prototype isolates of *O. tsutsugamushi* and illustrate their continued importance to research into scrub typhus. It is our aim in this review to verify the historic and genetic authenticity of three established prototype strains of *O. tsutsugamushi*, Gilliam, Karp, and Kato, and to examine the genetic integrity of the strains, which continue to be used in scrub typhus research. 

## 2. History of Scrub Typhus and *Orientia* Prototype Strains during and after World War II

As stated, scrub typhus played a significant role during WWII, but was little known outside Japan at the start of the war. With the onset of the war the American military found itself deployed to regions of the Far East and experiencing scrub typhus. Although the disease was well known even before WWII to British, Australian and Japanese investigators working in the region, US medical officers initially considered scrub typhus to be of minor military importance. The importance of the disease also seemed to be only partially understood by the Japanese military. Captured reports showed that Japanese medical officers in Burma recognized it in outbreaks of disease in Japanese Forces or European internees in Burma, Thailand, Malaya, Sumatra and Java [47]. However, they reported it as only showing similarities, but with milder infections, to the tsutsugamushi disease endemic to mainland Japan [48]. 

Based on the experience of the Allies during World War I and the anticipated wartime impact of typhus fever in Western Europe and North Africa, the United States of America Typhus Commission (USATC) was constituted on December 24, 1942 by Executive Order of President Roosevelt as a joint Army, Navy and Public Health Service mission [6,49,50]. It transpired that epidemic or louse-borne typhus was well controlled in the European Theater of Operations and had little impact on Allied forces. Though there were thousands of cases in the civilian sector, only 104 cases were reported in US troops for the entire war [47,50]. However, it soon became apparent to the commission that a different rickettsiosis in the Far East was having a significant, potentially mission compromising impact on deployed troops within the Asia-Pacific Theater of Operations. Subsequently members of the Commission, some of whom were already working in the European Theater in Cairo, were sent to the Far East to investigate. Complementary efforts were initiated between the United States Army Scrub Typhus Research Unit in Burma and the British Field Typhus Research Unit at Imphal, in eastern India [9]. Allied British, Australian and American military physicians and scientists, some trained in epidemiology, medical entomology and clinical microbiology worked to control major scrub typhus outbreaks among Allied troops from India to as far away as New Guinea and the Philippine Islands. At the end of the war, many of those medical officers continued their service and seized the opportunity to further investigate scrub typhus. The efforts of those researchers, both during the war, and soon thereafter together with scientists in Japan, allowed not only the better diagnosis of cases, but also led to the isolation and propagation of the obligate intracellular etiologic agent, currently named *O. tsutsugamushi*. Their work shows benefits to this very day [9,51,52]. With no effective antibiotic therapy available, the negative military impact of scrub typhus during WWII was indeed significant [17,36,53]. Approximately 18,000 scrub typhus cases were reported among allied servicemen from 1941 to 1945 with fatality rates as high as 35.3 per cent [47,54]. Diagnostics tools in the field were imprecise. There were no effective medical treatments, including the lack of effective antibiotics. Finally, preventive medical measures were limited in their ability to ameliorate the effect of the disease. In point of fact, in 2019, although there are effective treatments available, there is still no licensed vaccine to prevent scrub typhus [17,36,52,53]. 

By the end of WWII, scrub typhus had been shown to be a significant threat to members of the military within the Tsutsugamushi Triangle. Obviously, it also posed a serious medical threat to civilian inhabitants in this same region. Medical researchers were concerned with developing new strategies to diagnose and treat scrub typhus. Tools that emerged from the military medicine of WWII resulted in the development of procedures for the stable culture of strains of the agent of scrub typhus. These included improvements to the serial passage in mouse, hamster and guinea pig, development of culture in embryonated chicken eggs and sample transfer on dry ice [19,54]. The development of these techniques for research resulted in the availability of several established strains of *O. tsutsugamushi* gathered during or soon after WWII, including the Gilliam and Karp strains which would become generally accepted as prototype strains. 

Investigations immediately following the war by American and British scientists such as Dr. Joseph Smadel of the US Army Medical Department Research and Graduate School and Dr. J.R. Audy, of the British Army Scrub Typhus Research Laboratory, Institute for Medical Research (IMR), Kuala Lumpur, Malaysia (then, Malaya) were focused on the evaluation of antibiotic treatments for scrub typhus. The highly productive collaboration of those scientists and their parent institutions, initiated in March 1948, led to the establishment in 1953 of the United States Army Medical Research Unit-Malaysia, a laboratory component of the Walter Reed Army Institute of Research (WRAIR) [55]. In therapeutic antibiotic testing using a mouse model, two of the prototype *O. tsutsugamushi* strains described here, Gilliam and Karp, were among those used in the first successful evaluation of chloromycetin (chloramphenicol) [56]. Subsequently, Smadel, Audy and others were instrumental in using chloromycetin in the first successful antibiotic treatment of human scrub typhus [51,54,57]. Scrub typhus remains a disease that can be successfully treated when identified early in its course by antibiotics such as tetracyclines, chloramphenicol and azithromycin (although other compounds such as cephalosporins and ciprofloxacin are not efficacious). 

## 3. The Importance of Prototype Strains for the Study of Scrub Typhus

As mentioned, although a number of strains of *O. tsutsugamushi* have been maintained in culture and used by researchers, three strains stand out. These primary prototype strains are the Gilliam, Karp, and Kato strains. In the research describing the new genus *Orientia,* details are given that document the difference between strains of the new genus and the members of the genus *Rickettsia*, in which these strains had formerly been placed [45]. Data from the three prototype strains were vital in identifying differences between *Orientia* gen. nov. and *Rickettsia*. The Karp strain was designated the type strain of the new genus *Orientia*, while the sequences of the 16S rRNA genes of the Gilliam and Kato strains represented the primary initial molecular data which differentiated strains of scrub typhus from strains of *Rickettsia* [44]. The three strains were also those chosen when first determining the vital gene sequences that could be used to differentiate isolates of *O. tsutsugamushi* [43,58,59,60]. 

**Figure 1:**
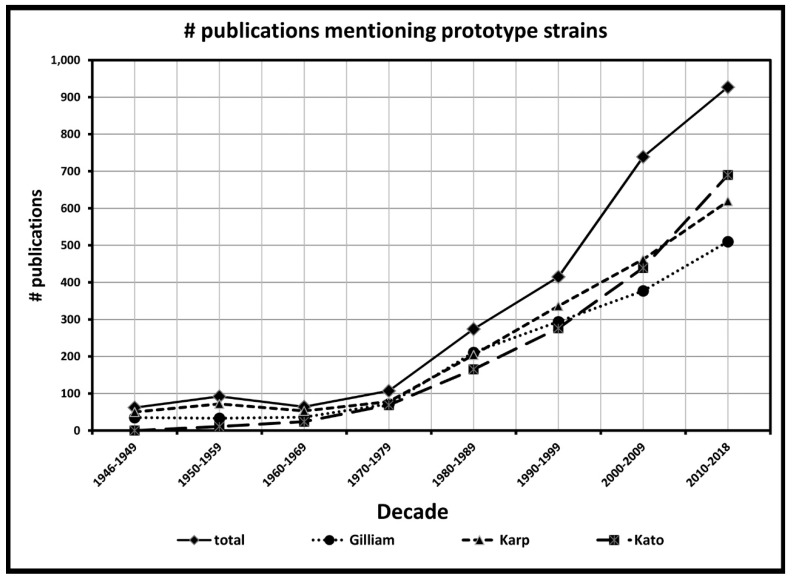
Number of scientific publications within each decade-long period that mention terms “tsutsugamushi” or “scrub typhus”, together with one or more of the prototype strain names “Karp”, “Gilliam” or “Kato”.

Increased medical relevance of scrub typhus, in general, and the prototype strain in particular, is given credence by the dramatic increase in citations in the research literature. We have used Google scholar to track the number of publications that mention the terms “scrub typhus” or “tsutsugamushi”. Through November 1, 2018, these terms appeared in 17,644 publications since the first mention of tsutsugamushi disease in the tracked literature in the 1890s, with 17,257 of these mentions occurring since the isolation of the prototype strains, Karp in 1943 and Gilliam in 1944. Google scholar was also used to track the number of publications that mention one or more of the three primary prototype strains (Gilliam, Karp and Kato) since the mid-1940s. The three prototype strains have been mentioned directly within the body of 2680 publications in that time, with the number of references increasing in recent decades. The increase over time is shown by the number of publications referring to the prototype strains within each decade, as shown in Figure 1. The substantial proportion (>15%) of all scrub typhus publications during the past 73 years that also refer to the use of the Gilliam, Karp or Kato prototype strains as baselines for studies of new and improved diagnostics, and treatments, or as tools to indicate the occurrence of scrub typhus indicates the importance of these strains to our knowledge of scrub typhus. In contrast to the number of times that Gilliam, Karp or Kato are mentioned in the literature, other strains that might be considered possible prototype strains, have been used substantially less. Only three strains have appeared over 500 times in the literature, Kawasaki (826), Boryong (536) and Ikeda (517). The latter two isolates represent the first two genome sequences of *Orientia*. Six other strains, Kuroki (363), Shimokoshi (186), TA763 (169), TA716 (161), Saitama (156) and TA686 (103) are mentioned over 100 times. 

The number of references to the three prototype strains increases and continues to increase substantially in each decade from 1980 onwards, as molecular techniques begin to be used for the study of *Orientia*. The prototype strains of *O. tsutsugamushi* have been used to develop diagnostic methods such as the polymerase chain reaction (PCR) and quantitative real-time or qPCR [61]. In the molecular study of *O. tsutsugamushi*, the first DNA gene sequences were published in 1990, coming from the prototype strains Karp [58,59] and Gilliam [60]. The first sequence from the Kato strain did not appear until 1992 [62]. Meanwhile, even as evidence of emerging antibiotic resistance and increased disease incidence continues to be reported, research on scrub typhus continues. 

## 4. The History of Three Prototype Strains of *O. tsutsugamushi*

### 4.1. Gilliam Strain

**Figure 2:**
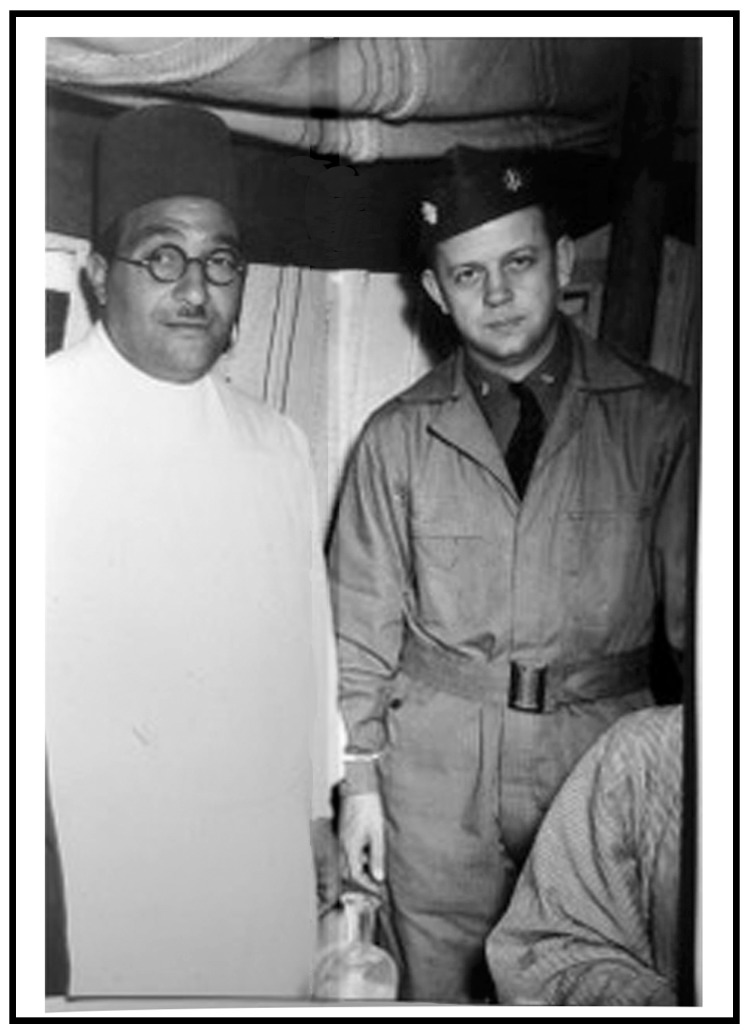
Lt. Col. Alexander Gilliam, 1943—in Cairo (Photo courtesy of Laura and Sandy Gilliam).

The Gilliam strain originated from the blood of Lt. Col. (Dr.) Alexander Gordon Gilliam, United States Public Health Service (USPHS) (B: 22 December 1904, D: December 12, 1963; pronounced “gillum”). Dr. Gilliam was a Senior Surgeon, a trained epidemiologist in the USPHS, and a member of the USATC [47,63]. Prior to the war, he had been active in polio research with the USPHS in Washington, DC, and, following the war, he worked on the Salk polio vaccine at the University of Michigan [64]. In January, 1943, he deployed to the typhus laboratory in Cairo, Egypt, to assist in a field trial evaluating the Cox-type epidemic typhus vaccine, no doubt becoming familiar with rickettsial isolation models and diagnosis [63]. Figure 2 shows Lt. Col. Gilliam during his deployment in Cairo in 1943. In September, 1943, he was posted to Chungking, China [64,65]. 

Late in 1943 when it was recognized that an outbreak had occurred of what was termed “CBI fever” (China–Burma–India Theater of Operations) in the Ledo (Assam) region, Lt. Col. Gilliam was posted to the region to assist with the investigation [7,66]. An 8.9% case fatality rate had been reported among United States troops in the India–Burma Theater [7]. In December, 1943, Gilliam and another medical officer, WS Jones, became ill and were diagnosed with scrub typhus [47,67]. Their infection was likely contracted while working in the “21 to 23” mile marker region of the Stilwell Road, where 66 cases had been reported in November and December, 1943 [68]. This area was considered to be a hotspot for scrub typhus, probably related to the occurrence of a “mite island” related to the clearance of secondary (“scrub”) forest for the construction of the Stilwell Road [69]. Both Gilliam and Jones were admitted to the 20th General Hospital, Marghuerita, (India) Assam in early January 1944, having become febrile 13 and 14 days respectively after entering the endemic area. As detailed in recent recorded interviews with Gilliam’s daughter (2013) and son (2014), Dr. Gilliam nearly died of the disease [64,65,70]. They relate that, in an example of the era of WWII medicine before the availability of effective antibiotics, their mother received a telephone call in January, 1944, telling her that her husband was ill with typhus and that he was likely to die. Nevertheless, Gilliam survived the disease, and returned home in March, 1944, but remained “sickly” and showed evidence of mental instability for several months thereafter. He had survived scrub typhus but took nearly a year to fully recover from his infection. Dr. Gilliam died of cancer in 1963.

**Figure 3:**
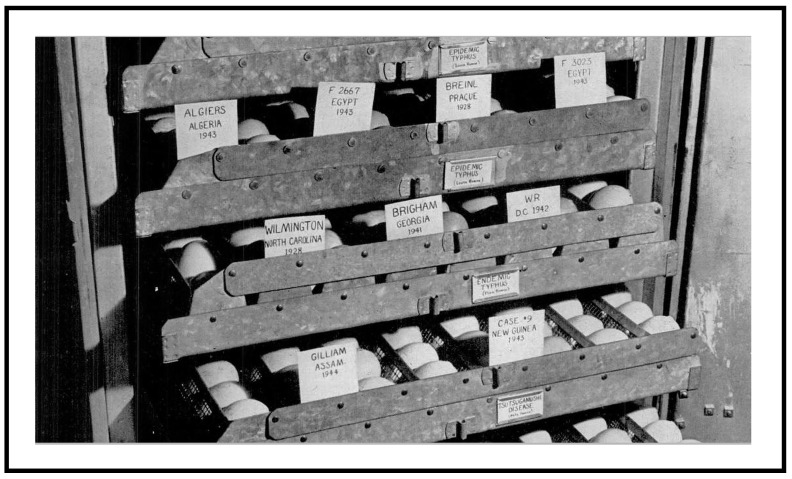
Egg cultures of Gilliam strain (lower left) maintained at the US National Institutes of Health, Bethesda Maryland in Spring 1944. Note, other rickettsial strains in culture including Breinl and Wilmington strain of *Rickettsia prowazekii* and *Rickettsia typhi*, respectively, and Case #9, a New Guinea isolate from a scrub typhus patient. Picture appeared in Life Magazine, May 1, 1944, page 65.

The passage history and disposition of the Gilliam strain, from the initial inoculation of a guinea pig with Dr. Gilliam’s blood in January, 1944, to the present, is relatively complete [21]. The original isolation and source information is noted on Walter Reed Army Medical Center (WRAMC) Form 543 maintained at WRAIR/Naval Medical Research Center (NMRC) as “1944-China–Burma–India Theater, from patient Dr. Gilliam.” Blood from the infected guinea pig was serially passed in guinea pigs and transported to the USNIH, Bethesda, MD, USA, received March, 1944. In *Crisis Fleeting* [66], Jones described a *Life Magazine* issue dated May 1, 1944, in which a photograph of an incubator at the United States National Institutes of Health (USNIH) showed an egg labeled “Gilliam”. It likely represented the culture of the isolate in question (as illustrated in the Life Magazine photo, above, Figure 3). The number of passages in guinea pigs prior to egg inoculation is unknown, but materials were serially passed in embryonated hen eggs upon receipt at the USNIH. In 1946, Dr. Norman H. Topping of the USNIH recorded the transfer of the Gilliam strain, as the 11th egg passage, to the Army Medical Service Graduate School, which in 1953 was renamed Walter Reed Army Institute of Research [71].

Records show continuous passages of the Gilliam strain at WRAIR, together with the other two prototype strains (Karp and Kato), until 1988 when the entire rickettsial inventory was transferred to the Rickettsial Diseases Department, Naval Medical Research Institute (NMRI), now the NMRC. This was part of the United States Department of Defense mandated consolidation of the Army and Navy Rickettsial Diseases Programs, completed in November, 1988. Gilliam strain passage information from a WRAIR publication of that era reads: Rtsu Gilliam, E164 (L-3) E 8. Note that “E” indicates egg passage and “L3” indicates triple plaque purification using murine L-cells to maintain genetic identity of the strain [72]. The three strains were initially provided to the NMRI by B.L. Elisberg and F.M. Bozeman, Department of Rickettsial Diseases, WRAIR. The Gilliam strain was originally deposited with the American Type Culture Collection or ATCC by Elisberg and Bozeman and assigned as ATCC VR-312. Stocks of ATCC VR-312 are no longer available from the ATCC, although a 1980s-era ATCC catalog lists Gilliam as “GP/3?, CE/133 (Lot#1)”. 

The relatively low virulence of Gilliam in mice following multiple passages prompted some to suggest the strain as a potential vaccine candidate [73,74,75]. However, the organism remained quite virulent for humans. For example, an anecdote provided by Dr. Charles Wisseman, Jr., a preeminent wartime (WWII) rickettsiologist and professor at the University of Maryland, underscored the virulence of the Gilliam strain in humans. During an open forum of a meeting of the American Society for Rickettsiology in which the strain was being discussed as a potential vaccine candidate he stated that it might not kill mice but that “I knew Gilliam and it damned near killed him.” [76]. In their early studies, Smadel et al. showed the Gilliam strain also to be quite toxic, relative to other strains of *O. tsutsugamushi* [71]. Compared to Karp strain, the Gilliam strain causes less severe disease in various mouse strains. However, the Gilliam strain versus the Karp strain in rhesus macaque scrub typhus model caused more severe clinical signs and overall induced more pronounced host immune responses similar to human scrub typhus [77]. Finally, the continued virulence of the strain was apparent. A military laboratory technician performing intravenous inoculation of the Gilliam strain into mice in the early 1980s became ill requiring hospitalization and antibiotic treatment [76]. Clearly, the isolation of the Gilliam strain from the blood of Alexander Gordon Gilliam and successful transport of the temperature labile isolate to Washington, DC, in that very difficult war-time environment has proven very useful for subsequent and extensive investigations of the disease.

### 4.2. Karp Strain

When WWII military operations began in the Southwest Pacific, medical officers were generally unaware of the disease that soon developed into a serious problem resulting in hundreds and later thousands of cases. In the summer of 1942, cases of scrub typhus were reported in Australian and American troops in regions of Papua and the Mandated Territory of New Guinea, including the Buna-Gona area [50]. In October 1943, USATC doctors arrived and began examining ongoing outbreaks, which included a pair of fatal cases. Their epidemiological studies developed several isolates including the Kostival and Buie strains [12]. Around that time, an American soldier named Karp who had been deployed in the Buna-Guna region of New Guinea was wounded and subsequently evacuated from the region to the 42nd US Hospital, Brisbane, Australia [19]. While hospitalized, he became febrile. He was diagnosed with scrub typhus and had been presumably infected while deployed in New Guinea. His collected blood was submitted to the Laboratory of Microbiology and Pathology, Queensland Health Dept. on 15 January, 1943. The blood clot was inoculated intraperitoneally into a guinea pig and at 10 days post inoculation, the febrile guinea pig was sacrificed, liver-spleen-kidney emulsions were prepared, then sequentially passed. During the multiple passes, rickettsiae appeared to become more virulent, killing about 50% of inoculated guinea pigs, an unusual finding for this organism in this animal model. The material also proved virulent in mice, killing them in 6 to 10 days and showing organisms upon staining of peritoneal fluid. The isolate was sent to Dr. F.M. Burnet, the future Nobel Prize recipient, at the Walter and Eliza Hall Memorial Institute for Medical Research, Melbourne, Australia, and was there passed again in guinea pigs. Tissues were provided to CDR I.L.V. Norman, MC, US Navy by Burnet for transport on dry ice to the United States. The initial dry ice shipment of infected tissues to the USA was unsuccessful, probably due to thawing during transit. On the second attempt, frozen samples were received at the NMRI, Bethesda MD USA on 17 August, 1943. The isolate was subsequently used in scrub typhus vaccine and immunological studies at NMRI [12,18,19,78]. Although the rickettsia did not kill soldier Karp, the strain subsequently infected or killed several laboratory workers including a close associate and collaborator of Burnet at the Hall Institute, Dora Lush [49,79].

Note that, as stated above, *O. tsutsugamushi* is very infectious, but is highly temperature labile, making it difficult to both transport and work with. It is unusual to have a strain virulent for guinea pigs. As with the Gilliam strain, records show continuous passages at WRAIR of the Karp strain until 1988 when the rickettsial inventory was transferred to the Rickettsial Diseases Department, NMRI. The Karp strain was deposited in the ATCC by H.S. Fuller and assigned as ATCC VR-150. The primary reason that elevated the Karp strain to its current status, and to its label as a prototype strain, is that it was the first isolate to become generally available for studies [19].

### 4.3. Kato Strain

According to Dr. Tsunehisa Suto [80], the Kato strain is purported to have been isolated from a classic human scrub typhus case of a febrile 15-year-old boy from Kurosawa village, Naka-Kanbara district, Niigata Prefecture, September 2, 1952. The culture then involved mouse inoculation of blood from the patient. The “1955 Professional Report” of the US Army 406th Medical General Laboratory in Japan describes the successful isolation of rickettsiae from two patients from Niigata Prefecture, including a positive OX-K Weil–Felix reaction to “Kato” strain infected mice. This is presumably the same Kato strain described by Shishido in 1958 [81] as “originally isolated from a patient of scrub typhus (tsutsugamushi disease) in Niigata Prefecture, Japan”. According to Dr. Suto, there were two passage histories, one at Niigata University, and a second documented at the Japanese National Institute of Health (JNIH), presumably from a subculture of the isolate at Niigata University transferred prior to 1957. The Kato strain has been retained in labs at the JNIH. Documentation indicates that the Kato strain was subsequently passed to a laboratory in the USA, specifically the WRAIR lab of Elisberg, in 1964 [82]. It was from this subculture that Elisberg and Bozeman deposited the strain to ATCC, where it has been assigned as ATCC VR-609.

Finally, it is important to note that in molecular studies published in the early 1990s from Japanese labs, which are discussed in the next section, mention is clearly made that material from all three prototype strains was obtained from cultures maintained at the JNIH, apparently since the mid-1950s. This indicates that parallel culture of the prototype strains in the United States and Japan have been present for over 50 years without any indication that the cultures were transferred between the two main repositories since that time. 

## 5. Molecular Comparison of Parallel Samples of Prototype Strains

When considering the use of a prototype strain in setting benchmarks for research, several uncertainties can be raised. One can first question whether each particular application of a prototype strain in new research truly involves the use of the strain, or whether problems such as contamination, mislabeling or other errors could result in the inadvertent use of an alternative unidentified strain. A second question relates to whether genetic changes, mutations, have occurred in a prototype strain over time while the strain has been passaged at a research center, such as the NMRC, a culture center, such as ATCC, or in a lab that uses the prototype strain. Such changes could result in the attenuation of an isolate, making it easier to culture, or less pathogenic. An example of such a change is well known, the Madrid E strain of *Rickettsia prowazekii*. That strain originated from a 1941 isolate from a case of typhus treated during an outbreak in Madrid, Spain. The isolate was passed routinely in eggs. A change abruptly occurred in culture, yielding reduced virulence for guinea pigs, first noted during the 11th passage. Work done with this strain of *R. prowazekii*, initially by Clavero and Perez Gallardo and later by Fox and associates, resulted in the development of the attenuated vaccine strain E [83]. If changes in genes of the *O. tsutsugamushi* prototype strains have occurred since the origin of the prototype strain, they could theoretically be tracked back, using the tools of molecular biology, to some point in time, and/or their subsequent dispersal to various centers or labs. 

Both of these questions can be addressed, at least partially, by comparing DNA sequences that have been deposited by various laboratories into the international DNA sequence repositories such as GenBank [84]. As previously mentioned, some DNA sequences for *O. tsutsugamushi* prototype strains have been reported and deposited since the early 1990s. Duplicate deposits were made for a number of these genes, sequences having been determined independently by different labs. In addition, in the genome era of the past few years (characterized here by the introduction of next-generation sequencing (NGS) technology), four labs have independently obtained either complete or nearly complete genome sequences from the three prototype strains, Gilliam, Karp and Kato. 

In our genetic analysis of prototype strains over time, we have compared the sequences of the following genes: the 56-kD TSA gene, the 47-kD membrane protease (*htr*A) gene, the 60-kD GroEL chaperonin gene, and the 16S rRNA gene (*rrs*). In each case, some DNA sequences were obtained from a prototype strain prior to the genome era, often by two independent laboratories. These sequences can be compared with one another and can then also be compared with the genome sequences from the four sequencing groups for the Karp strain and three each for the Gilliam and Kato strains. The results can be used to test whether the sequences obtained from putatively identical samples of a strain will yield comparisons that are within the limits of expected sequencing error, or whether any error has occurred in labeling the prototype strain. In some cases, these comparisons can also indicate whether any changes have occurred over time, at least from the 1990s and, in some cases, since the mid-1950s as the strains remained in culture.

Sequences for the four genes were downloaded, for older sequences, directly from the nucleotide database GenBank, or, for genome sequences, extracted from the NCBI whole genomic sequences (WGS) or sequence read archive (SRA) databases. Sequences were aligned using the alignment subroutine of MEGA6 [85]. The aligned sequences were then analyzed for pairwise sequence differences, as computed within MEGA6. Information about the sequences used for the analysis is provided in Supplement 1. 

### Results of Gene Comparisons:

In pairwise comparisons of the genome sequences for the four genes, all genome sequences for four duplicate genomes of Karp, and the three duplicate genomes from Kato, were identical (Tables S1–S4). For comparisons of the genome sequences of the Gilliam strain, pairwise comparisons of the three replicate genomes were identical for the 56-kD TSA gene, the 47-kD HtrA gene, and the 16S rRNA gene. The sequence of the 60-kD GroEL chaperonin gene from the LANO genome (contig LANO01000026) differed by a single nucleotide from the other two genome sequences (Table S3). The conclusion from these comparisons is that all genome sequencing groups appear to have valid duplicate samples of the three prototype strains. 

When comparing the genome sequences with earlier pre-genome sequences deposited in the DNA databases, some differences were observed. The most striking difference occurs for the 56-kD TSA gene from the Karp strain (Table S1). Here, the original sequence of the gene reported in 1990 (acc# M33004) differs from the genome sequences and from the other two pre-genome sequences by the presence of two in/del changes in the sequence that result in six nucleotide differences in the pair-wise aligned comparisons with the genome sequences. The changes would also alter a small segment of the amino acid sequence of the protein. The other two pre-genome sequences of the 56-kD TSA gene for Karp (acc#’s AY956315 and AY283180) each differ from the genome sequences by two nucleotide changes, but each of the latter pre-genome sequences differ at unique sites. The differences in these latter two pre-genome sequences are most likely to be simple sequencing errors. The differences observed for the original Karp sequences (acc# M33004) may be more complicated. The sequence differences may simply be due to sequencing errors from 1990s technology. However, the unique in/del changes in M33004 appear to be shared with several non-prototype isolates deposited in the DNA databases, although M33004 is not identical to any other sequence in the database. Are the differences simply pre-genome sequencing errors, or do they represent the presence, at least in the early stage of the analysis of scrub typhus molecular biology, of an alternative “Karp” strain in culture? The answer is equivocal, since DNA from the specific culture used for the original Karp 56-kD TSA sequence is unlikely to be available [58]. 

For comparisons of the pre-genome sequences of the 47-kD HtrA gene, all pre-genome sequences showed some differences from the genome sequences (Table S2). Each of the three pre-genome sequences (acc#’s L31933, L31934, and L11697) was produced from the same laboratory, and showed four differences from Karp genomes, four differences from Gilliam genomes and two differences for Kato genomes. It appears likely to us that all differences represent simple sequencing errors. 

For the 60-kD GroEL gene segment, the pre-genome sequences of Karp and Gilliam are identical to the genomes sequences, while the Kato pre-genome sequence differs from genome sequences at two sites, most likely representing simple sequencing errors (Table S3). 

Finally, for the 16S rRNA gene, pre-genome sequences from two labs are identical to genome sequences for Karp, sequences from three labs are identical to genome sequences for Gilliam, and sequences from two labs differ from each other by one nucleotide, with one of the sequences (acc# D38624) agreeing completely with the genome sequences (Table S4). 

The 16S rRNA gene sequences have additional importance since they indicate that isolates that have been separated since the 1950s were either identical or showed only a single difference (probably a sequencing error) from each other and from the genome sequences. In fact, the 16S rRNA gene sequence (acc # D38624) that was identical to genome sequences represents the isolate traceable to the JNIH and therefore represents the longest JNIH separation time between different versions of the same prototype strain. Likewise, the pre-genome era sequence of the Gilliam 56-kD TSA from Japan (acc # M33267, [60]) was identical with genome sequences that had been separated from it by culture in the United States at least since the 1950s. 

The sequence stability that we have observed over time for genes in the three prototype strains does not necessarily indicate that the genomes, or the sequences of genes between genomes of different *O. tsutsugamushi* isolates, are essentially genetically stable. Information from comparison of complete genomes indicates that the order of genes in different isolates may be quite unique [46]. While there are “islands” of core genes that have similar gene order in various isolates, the order of these “islands” between the genomes of individual isolates of *O. tsutsugamushi* is variable. We have compared the gene sequence order of duplicate genome sequences of the three prototype strains obtained from assorted laboratories that have been deposited in the international DNA databases. In general, these comparisons indicated that the orders of genes between the duplicate versions of a prototype genome are the same. Our confidence in these results must be tempered by the fact that only the Batty et al., 2018 [46], study used methodologies that could easily span long ranges of sequence that include repetitive elements. Additional comparisons of genomes sequences collected using methods that provide long sequence reads are required to provide high confidence in our conclusions. Further, the study of whether gene order might change between multiple time periods for a specific isolate has certainly not been done. 

The importance of intra- versus inter-genomic recombination for *Orientia* is a vital area of interest for our understanding of scrub typhus. Results from comparison of variation in the position of “islands” of core genes within the genomes of different isolates of *O. tsutsugamushi* [46] suggest that genome stability between isolates, may be affected by forces that have greater importance within the *Orientia* pan-genome than in most other bacterial taxa [86,87]. *Orientia* genomes appear to have been affected by intra- and inter-genome recombination, facilitated by repetitive sequences that are more abundant within the genome of *Orientia* than in most bacteria [88]. These processes can result in the scrambling of gene order within the genome and appear to have been responsible for potential horizontal transfer of gene segments, by recombination, between strains [87,89]. It should be noted that such recombination has only been inferred, not directly observed experimentally. The result, however, is that any strain of *Orientia* will represent a mosaic of gene histories, with each gene or block of genes, having a history that may be partially or substantially independent of the history for other genes. This mosaic will be as true of the prototype strains as it would be for any new strain isolated in the 2020s. The history and stability of genes, on the other hand, are not affected by these recombinational forces. Our studies, indicating the genetic stability of isolates over short evolutionary time periods is not in conflict with the picture of the dynamic genome mosaic. The fact that the order of genes within gene “islands” of duplicate versions of a prototype strain appears to remain constant is also not in conflict with a mosaic genome. The prototype isolates in culture do not have any opportunity to interact with other isolates, so there is no opportunity for inter-isolate genomic recombination. Whether, and how frequently, intra-cellular recombination might occur is currently unknown. The genes themselves are stable, indicating that there is no hypermutability at the nucleotide level. Each prototype strain represents the culmination of a unique evolutionary history made up of a mosaic of individual gene histories. Much information is still to be gleaned from genome analysis of the prototype strains and comparisons with each other and with the genomes of other strains from nature. This is an area of unique importance since the question remains unanswered as to exactly how the tremendous variability seen in isolates from nature progresses [40,90,91,92]. This question must be addressed if an effective vaccine can be developed. 

## 6. Discussion/Conclusions

We have documented the continuity of three prototype strains of *O. tsutsugamushi* that serve today as the basis of continued work on scrub typhus. Why are prototype strains important? Comparisons among strains using the established prototypes as baselines permit validation of new assays, both serological and molecular. Our review shows that the Gilliam, Karp and Kato strains that have been used for over 70 years remain stable and consistent. New findings on scrub typhus can be confidently grounded in the foundation that these strains have provided. The development of new diagnostic procedures can be founded on a stable base. The potential development of vaccines may ultimately be an area where prototype strains are important because they have failed to provide a consistent, successful product. Here, the prototypes are important because they can provide information about the heterogeneity of *O. tsutsugamushi* in nature. 

We believe the continued use of the established prototype strains and the inclusion of other unique strains into research and product development to be very important. The initial recovery and successful maintenance of the prototype strains, often under difficult war-time conditions, is nothing short of amazing. We have shown that the maintenance and genetic fidelity of those early strains has been successful. The identity of these strains has been maintained through hundreds of passages of eggs, animals and cell cultures. 

Serological testing of new isolates recovered from patients, rodents, and vector mites by complement fixation, direct and indirect fluorescent antibody testing and, more recently DNA sequence analysis indicate that the scrub typhus rickettsiae isolated from natural sources are highly variable, especially for those genes that are thought to be most important in eliciting immunological responses to infection [1,91,92]. Comparisons using the established prototypes as baselines permit validation of the new assays. 

From a phylogenetic framework, information for the prototype strains allows a context to be formed for more divergent taxa, which may be associated with *O. tsutsugamushi*. These include rare divergent strains such as the Shimokoshi strain, isolated in Japan [62]. The prototypes also put in context newly recognized taxa such as “*Candidatus* Orientia chuto”, isolated in Australia from a patient believed to be infected in the United Arab Emirates, as well as currently unnamed forms that have been reported to exist in Africa, South America and possibly even Europe [2]. 

The history of these agents puts into context the sociological conditions that led to isolation and study of the agents, warfare and medicine [93]. The stories of the Karp and Gilliam isolates indicate the importance of military medicine to our understanding of disease. For scrub typhus, information about the disease was scattered and unfocused until the occurrence of outbreaks in WWII caused the military to respond. 

The faithful curation of the collections of these fastidious organisms, including the willingness to maintain and share genetically consistent pure cultures, is essential to the ongoing research of the rickettsiae. In terms of the genetically hypervariable *O. tsutsugamushi*, these collections are necessary to address difficult questions such as how such diversity originated and how it is maintained in nature, given that their trombiculid vector feeds but once in its lifecycle and appears not to stably acquire and maintain new strains in contrast to other vector-parasite pairs, such as mosquitoes and the malaria parasite. The continued collection and maintenance of older strains, as well as the acquisition of new strains, will allow us to develop and test effective vaccines, develop new detection and identification systems, and control this hypervariable human disease. 

Additional information on the background of these and other isolates, and on general topics of scrub typhus is available at https://u.osu.edu/scrubtyphus. 

## Supplementary Materials

The following are available online at https://www.mdpi.com/2414-6366/4/2/75/s1, Supplement 1: Sources of DNA Sequences, Table S1: Pairwise comparisons of 56-kD TSA gene sequences, Table S2: Pairwise comparisons of 47-kD HtrA gene sequences, Table S3: Pairwise comparisons of GroEL chaperonin gene, Table S4: Pairwise comparisons of 16S rRNA gene sequences.
