# Supplementary material for: Origins, Importance and Genetic Stability of the Prototype Strains Gilliam, Karp and Kato of Orientia tsutsugamushi
Source: Trop Med Infect Dis. 2019 Apr 30;4(2):75. doi: 10.3390/tropicalmed4020075 (PMC6631653; doi:10.3390/tropicalmed4020075)
Supplement: Supplementary file 1 [file tropicalmed-04-00075-s001.zip › TMID Prototype strains revision-suppl 1.pdf]

Supplement 1. Sources of DNA Sequences:

Sources include GenBank files for either genome or gene sequences. Information has been retrieved for information on four genes: (1) the 56-kD TSA gene, (2) the 56-kD TSA gene, (3) the GroEL gene, and (4) 16S rRNA gene.

For all four gene comparisons, the genome sequences are as follows:

(a) Karp strain: WGS files LYMA02 [1], NZ_LANM00000000 [2], NZ_LS398548 [1], and the SRA files of genome project SRX1761260 [3];

(b) Gilliam strain: WGS files LS398551 [1], NZ_LANO00000000 [2] and from the SRA files of genome project SRX477935 [3];

    Note that the 47kDa (*htrA*) gene sequence is "missing" in one of the three deposited WGS sequence for the Gilliam strain (NZ_LANO01000000), but could be retrieved from the SRA files associated with that genome study.

(c) Kato strain: WGS files NZ_LS398550 [1], NZ_LANN00000000 [2], and the SRA files of genome project SRX1761385 [3].

For gene sequences, the sources are as follows:

(a) For the 56-kD TSA gene:
   (i) Gilliam strain: two pre-genome sequences, GenBank accession #'s M33267 [4] and DQ485289 [5]
   (ii) Karp strain: three pre-genome sequences, M33004 [6], AY956315 [7] and AY283180 [8]
   (iii) Kato strain: two pre-genome sequences M63382 [9] and AY836148 for Kato [109]

(b) For 47kDa (*htrA*) gene:
   (i) Gilliam strain: one pre-genome sequences, GenBank accession # L31934 [11]
   (ii) Karp strain: one pre-genome sequences, L31933 [11]
   (iii) Kato strain: three pre-genome sequences L11697 [12], and HM595492 and HM595493 [13].

(c) For the GroEL gene:
   (i) Gilliam strain: the sequences of only a portion of one version of the gene (546 nucleotides out of 1668) were determined, accession # AY191585 [14]
   (ii) Karp strain: one pre-genome sequences, M31887 [15]
   (iii) Kato strain: two pre-genome sequences, JX188393 [16], and only a portion of one version of the gene (546 nucleotides out of 1668) AY191586 [14].

(d) For the 16S rRNA gene:
   (i) Gilliam strain: three pre-genome sequences, U17256 [17], D38622 [18] and L36222 [197].
   (ii) Karp strain: two pre-genome sequences, U17257 [17] and D38623 [18],
   (iii) Kato strain: two pre-genome sequences, U17258 [17] and D38624 [18]

The pre-genome sequences for the 16S rRNA gene for all three prototype strains, and the 56kD TSA gene from Gilliam are particularly noteworthy because they represent samples of the isolates that have presumably been separated from each other, or from genome sequences since the mid-1950's, one sample representing laboratory passages in the United States (primarily at NMRC and its previous incarnations) and the second representing laboratory passages at the JNIH in Tokyo and subsequent culturing at Niigata University.

Data References:

1. Batty, E.M.; Chaemchuen, S.; Blacksell, S.; Richards, A.L.; Paris, D.; Bowden, R.; Chan, C.; Lachumanan, R.; Day, N.; Donnelly, P.; Chen, S.; Salje, J. Long-read whole genome sequencing and comparative analysis of six strains of the human pathogen *Orientia tsutsugamushi*. *PLOS Negl. Trop. Dis.* **2018**, *12*, e0006566.
2. Daugherty, S.C.; Su, Q.; Abolude, K.; Beier-Sexton, M.; Carlyon, J.A.; Carter,R.; Day, N.P.; Dumler, S.J.; Dyachenko, V.; Godinez, A.; Kurtti, T.J.; Lichay, M.; Mullins, K.E.; Ott, S.; Pappas-Brown, V.; Paris, D.H.; Patel, P.; Richards, A.L.; Sadzewicz, L.; Sears, K.; Seidman, D.; Sengamalay, N.; Stenos, J.; Tallon,L. J.; Vincent, G.; Fraser, C.M.; Munderloh, U.; Dunning-Hotopp, J.C. Genome Sequencing of Rickettsiales. **2015**, Unpublished.
3. Fleshman, A.; Mullins, K.; Sahl, J.; Hepp, C.; Nieto, N.; Wiggins, K.; Hornstra, H.; Kelly, D.; Chan, T.C.; Phetsouvanh, R.; Dittrich, S.; Panyanivong, P.; Paris, D.; Newton, P.; Richards, A.; Pearson, T. Comparative pan-genomic analyses of *Orientia tsutsugamushi* reveal an exceptional model of bacterial evolution driving genomic diversity. *Microbial Genomics* **2018,** *4.*
4. Ohashi, N.; Nashimoto,H.; Ikeda,H.; Tamura, A. Diversity of immunodominant 56-kDa type-specific antigen (TSA) of *Rickettsia tsutsugamushi*. Sequence and comparative analyses of the genes encoding TSA homologues from four antigenic variants. *J. Biol. Chem*. **1992**, *267*, 12728-12735.
5. Tamura , A.; Ohashi, N.; Urakami, H.; Miyamura, S. Classification of *Rickettsia tsutsugamushi* in a new genus, *Orientia* gen. nov., as *Orientia tsutsugamushi* comb. nov. *Int. J. Syst. Bacteriol*. **1995**, *45*, 589-591.
6. Stover, C.; Marana, D.; Carter, J.; Roe, B.; Mardis, E.; Oaks, E. The 56-kilodalton major protein antigen of *Rickettsia tsutsugamushi*: Molecular cloning and sequence analysis of the sta56 gene and precise identification of a strain-specific epitope. *Infect. Immun.* **1990**, *58*, 2076–2084.
7. Huang, I.T.; Yang, H.H.; Lin, C.H.; Huang, S.T.; Huang, J.H.; Chen, L.K. **2005**. Unpublished.
8. Lai, Y.D.; Zheng, X.Y.; Zhan,X.M. Gene cloning and sequencing the 56 kilodalton major protein antigen of *Orientia tsutsugamushi*, scrub typhus. **2003.** Unpublished.
9. Ohashi,N.; Nashimoto,H.; Ikeda,H.; Tamura, A. Diversity of immunodominant 56-kDa type-specific antigen (TSA) of *Rickettsia tsutsugamushi*. Sequence and comparative analyses of the genes encoding TSA homologues from four antigenic variants. *J. Biol. Chem*. **1992**, *267*, 12728-12735.
10. Yang, H.H.; Huang, I.T.; Huang, J.H.; Chen, L.K. *Orientia tsutsugamushi* new strain Taiwan CDC Kato 56-kDa type-specific antigen gene. **2004**. Unpublished.
11. Kim,I.-J. **1996.** Unpublished.
12. Kim,I.-J.; Kim, I.-S.; Choi, I.-H.; Chang, W.-H.; Choi, M.-S. Characterization of gene for 47Kda protein of Rickettsia tsutsugamushi. **1994.** Unpublished.
13. Jiang, J.; Paris, D.H.; Blacksell, S.D.; Aukkanit, N.; Newton, P.N.; Phetsouvanh, R.; Izzard, L.; Stenos, J.; Graves, S.R.; Day, N.P.; Richards, A.L. Diversity of the 47-kD HtrA nucleic acid and translated amino acid sequences from 17 recent human isolates of *Orientia*. *Vector Borne Zoonotic Dis.* **2013** *13 (6),* 367-375.
14. Lee, J.H.; Park, H.S.; Jang, W.J.; Koh, S.E.; Kim, J.M.; Shim, S.K.; Park, M.Y.; Kim, Y.W.; Kim, B.J.; Kook, Y.H.; Park, K.H.; Lee, S.H. Differentiation of rickettsiae by groEL gene analysis. *J. Clin. Microbiol*. **2003**, *41,* 2952-2960.
15. Stover, C.K.; Marana, D.P.; Dasch, G.A.; Oaks, E.V. Molecular cloning and sequence analysis of the Sta58 major antigen gene of *Rickettsia tsutsugamushi*: sequence homology and antigenic comparison to the 60-kilodalton family of stress proteins. *Infect. Immun.* **1990**, *58*, 1360–1368.
16. Arai, S.; Tabara, K.; Yamamoto, N.; Fujita, H.; Itagaki, A.; Kon, M.; Satoh, H.; Araki, K.; Tanaka-Taya, K.; Takada, N.; Yoshikawa, Y.; Ishihara, C.; Okabe, N.; Oishi, K. Molecular phylogenetic analysis of *Orientia tsutsugamushi* based on the groES and groEL genes. *Vector Borne Zoonotic Dis*. **2013,** *11*, 825-829.
17. Stothard, D.R.; Fuerst, P.A. Evolutionary analysis of the spotted fever and typhus groups of *Rickettsia* using 16S rRNA gene sequences. *Systematic and Applied Microbiology* **1995,** *18*, 52-61.
18. Tamura, A.; Urakami, H.; Ohashi, N. A comparative view of *Rickettsia tsutsugamushi* and other groups of rickettsiae. *Euro. J. Epidemiol.* **1991**, *259-269.*
19. Roux, V.; Raoult, D. Phylogenetic analysis of the genus Rickettsia by 16S rDNA sequencing. *Res. Microbiol.* **1995,** *146*, 385-396.
