# Supplementary material for: Origins, Importance and Genetic Stability of the Prototype Strains Gilliam, Karp and Kato of Orientia tsutsugamushi
Source: Trop Med Infect Dis. 2019 Apr 30;4(2):75. doi: 10.3390/tropicalmed4020075 (PMC6631653; doi:10.3390/tropicalmed4020075)
Supplement: Supplementary file 1 [file tropicalmed-04-00075-s001.zip › TMID Prototype strains revision-Suppl Table 1REV.pdf]

		1	2	3	4	5
1	M33267-Gilliam	-	1575	1575	1575	1575
2	DQ485289_taiwan_CDCGilliam	3	-	1575	1575	1575
3	LANO01000035_Gilliam	0	3	-	1575	1575
4	LS398551_Gilliam	0	3	0	-	1575
5	SRX1761334_Gilliam	0	3	0	0	-

		1	2	3	4	5	6	7
1	M33004-Karp	-	1600	1600	1600	1600	1600	1600
2	AY956315-CDCKarp	7	-	1596	1596	1596	1596	1596
3	AY283180-Karp	8	4	-	1596	1596	1596	1596
4	LANM-Karp	6	2	2	-	1596	1596	1596
5	LYMA-Karp	6	2	2	0	-	1596	1596
6	LS398548 -Karp	6	2	2	0	0	-	1596
7	SRX1761389-Karp	6	2	2	0	0	0	-

		1	2	3	4	5
1	M63382-Kato	-	1587	1587	1587	1587
2	AY836148-CDCKato	2	-	1587	1587	1587
3	LANN_Kato PP	0	2	-	1587	1587
4	LS398550-Kato	0	2	0	-	1587
5	SRX1761385 Kato	0	2	0	0	-

pre-genome x pre-genome

pre-genome x genome

genome x genome

Supplementary Table 1. Pairwise comparisons of 56-kD TSA gene sequences of *Orientia tsutsugamushi* prototype strains Gilliam, Karp, and Kato maintained in separate laboratories, including gene sequences published in the early 1990's (pre-genome) and complete or near-complete genome sequences (genome).
