# Supplementary material for: Origins, Importance and Genetic Stability of the Prototype Strains Gilliam, Karp and Kato of Orientia tsutsugamushi
Source: Trop Med Infect Dis. 2019 Apr 30;4(2):75. doi: 10.3390/tropicalmed4020075 (PMC6631653; doi:10.3390/tropicalmed4020075)
Supplement: Supplementary file 1 [file tropicalmed-04-00075-s001.zip › TMID Prototype strains revision-Suppl Table 2REV.pdf]

		1	2	3	4
1	L31933-Gm47-Gilliam	-	1401	1401	1401
2	LS398551_Gilliam	4	-	1401	1401
3	SRX477935_Gilliam	4	0	-	1401
4	SRX1761334_Gilliam	4	0	0	-

		1	2	3	4	5
1	L31934-Kp47	-	1401	1401	1401	1401
2	LS398548_Karp	4	-	1401	1401	1401
3	LYMA02000066_Karp	4	0	-	1401	1401
4	LANM01000009_Karp	4	0	0	-	1401
5	SRX1761260_Karp	4	0	0	0	-

		1	2	3	4	5	6
1	L11697-Pkt5-Kato	-	1401	1401	1401	1401	1401
2	HM595492_Kato_HtrA_(htrA)	2	-	1401	1401	1401	1401
3	HM595493_Kato_HtrA_(htrA)	2	0	-	1401	1401	1401
4	LANN01000016_Kato_PP	2	0	0	-	1401	1401
5	LS398550_Kato	2	0	0	0	-	1401
6	SRX1761385_kato	2	0	0	0	0	-

pre-genome x pre-genome

pre-genome x genome

genome x genome

Supplementary Table 2. Pairwise comparisons of 47-kD HtrA gene sequences of *Orientia tsutsugamushi* prototype strains Gilliam, Karp, and Kato maintained in separate laboratories, including gene sequences published in the early 1990's (pre-genome) and complete or near-complete genome sequences (genome).
