# Supplementary material for: Origins, Importance and Genetic Stability of the Prototype Strains Gilliam, Karp and Kato of Orientia tsutsugamushi
Source: Trop Med Infect Dis. 2019 Apr 30;4(2):75. doi: 10.3390/tropicalmed4020075 (PMC6631653; doi:10.3390/tropicalmed4020075)
Supplement: Supplementary file 1 [file tropicalmed-04-00075-s001.zip › TMID Prototype strains revision-Suppl Table 3REV.pdf]

		1	2	3	4	5
1	M31887_Karp	-	1668	1668	1668	1668
2	LYMA02000027_Karp	0	-	1668	1668	1668
3	LANM01000016_Karp	0	0	-	1668	1668
4	LS398548_Karp	0	0	0	-	1668
5	SRX1761260_Karp	0	0	0	0	-

		1	2	3	4
1	AY191585_Gilliam	-			
2	LANO01000026_Gilliam	0	-		
3	LS398551_Gilliam	0	1	-	
4	SRX1761334_Gilliam	0	1	0	-

		1	2	3	4	5
1	AY191586_Kato	-	546	546	546	546
2	JX188393_Kato_GroESL	2	-	1668	1668	1668
3	LANN01000021_Kato	2	0	-	1668	1668
4	LS398550_Kato	2	0	0	-	1668
5	SRX1761385_kato	2	0	0	0	-

pre-genome x pre-genome

pre-genome x genome

genome x genome

Supplemental Table 3. Comparison among duplicate sequences of GroEL chaperonin gene between prototype strains. Above diagonal = length of sequence comparison; below diagonal = number of nucleotide differences between paired sequences.

		1	2	3	4
1	AY191585_Gilliam	-			
2	LANO01000026_Gilliam	0	-		
3	LS398551_Gilliam	0	1	-	
4	SRX1761334_Gilliam	0	1	0	-

		1	2	3	4	5
1	M31887_Karp	-	1668	1668	1668	1668
2	LYMA02000027_Karp	0	-	1668	1668	1668
3	LANM01000016_Karp	0	0	-	1668	1668
4	LS398548_Karp	0	0	0	-	1668
5	SRX1761260_Karp	0	0	0	0	-

		1	2	3	4	5
1	AY191586_Kato	-	546	546	546	546
2	JX188393_Kato_GroESL	2	-	1668	1668	1668
3	LANN01000021_Kato	2	0	-	1668	1668
4	LS398550_Kato	2	0	0	-	1668
5	SRX1761385_kato	2	0	0	0	-

pre-genome x pre-genome

pre-genome x genome

genome x genome

Supplementary Table 3. Pairwise comparisons of 60-kD GroEL gene sequences of *Orientia tsutsugamushi* prototype strains Gilliam, Karp, and Kato maintained in separate laboratories, including gene sequences published in the early 1990's (pre-genome) and complete or near-complete genome sequences (genome).
