# Supplementary material for: Origins, Importance and Genetic Stability of the Prototype Strains Gilliam, Karp and Kato of Orientia tsutsugamushi
Source: Trop Med Infect Dis. 2019 Apr 30;4(2):75. doi: 10.3390/tropicalmed4020075 (PMC6631653; doi:10.3390/tropicalmed4020075)
Supplement: Supplementary file 1 [file tropicalmed-04-00075-s001.zip › TMID Prototype strains revision-Suppl Table 4REV.pdf]

		1	2	3	4	5	6
1	U17257_Karp	-	1453	1453	1453	1453	1453
2	D38623_Karp	0	-	1459	1459	1459	1459
3	LANM01000019_Karp	0	0	-	1480	1480	1480
4	LYMA02000036_Karp	0	0	0	-	1480	1480
5	LS398548_Karp	0	0	0	0	-	1480
6	SRX1761260_Karp	0	0	0	0	0	-

		1	2	3	4	5	6
1	U17256_Gilliam	-	1440	1457	1457	1457	1457
2	L36222_Gilliam	0	-	1440	1448	1448	1448
3	D38622_Gilliam	0	0	-	1459	1459	1459
4	LANO01000030_Gilliam	0	0	0	-	1501	1501
5	LS398551_Gilliam	0	0	0	0	-	1501
6	SRX1761334_Gilliam	0	0	0	0	0	-

		1	2	3	4	5	6
1	U17258_Kato	-	1459	1459	1459	1459	1459
2	D38624_Kato	1	-	1459	1459	1459	1459
3	LANN01000025_Kato_PP	1	0	-	1501	1501	1501
4	LY337763_Kato	1	0	0	-	1501	1501
5	LS398550_Kato	1	0	0	0	-	1501
6	SRX1761385_kato	1	0	0	0	0	-

pre-genome x pre-genome

pre-genome x genome

genome x genome

Supplemental Table 4. Comparison among duplicate sequences of 16S rRNA gene between prototype strains. Above diagonal = length of sequence comparison; below diagonal = number of nucleotide differences between paired sequences.

		1	2	3	4	5	6
1	U17256_Gilliam	-	1440	1457	1457	1457	1457
2	L36222_Gilliam	0	-	1440	1448	1448	1448
3	D38622_Gilliam	0	0	-	1459	1459	1459
4	LANO01000030_Gilliam	0	0	0	-	1501	1501
5	LS398551_Gilliam	0	0	0	0	-	1501
6	SRX1761334_Gilliam	0	0	0	0	0	-

		1	2	3	4	5	6
1	U17257_Karp	-	1453	1453	1453	1453	1453
2	D38623_Karp	0	-	1459	1459	1459	1459
3	LANM01000019_Karp	0	0	-	1480	1480	1480
4	LYMA02000036_Karp	0	0	0	-	1480	1480
5	LS398548_Karp	0	0	0	0	-	1480
6	SRX1761260_Karp	0	0	0	0	0	-

		1	2	3	4	5	6
1	U17258_Kato	-	1459	1459	1459	1459	1459
2	D38624_Kato	1	-	1459	1459	1459	1459
3	LANN01000025_Kato_PP	1	0	-	1501	1501	1501
4	LY337763_Kato	1	0	0	-	1501	1501
5	LS398550_Kato	1	0	0	0	-	1501
6	SRX1761385_kato	1	0	0	0	0	-

pre-genome x pre-genome

pre-genome x genome

genome x genome

Supplementary Table 4. Pairwise comparisons of 16S rRNA gene sequences of *Orientia tsutsugamushi* prototype strains Gilliam, Karp, and Kato maintained in separate laboratories, including gene sequences published in the early 1990’s (pre-genome) and complete or near-complete genome sequences (genome).
